# Hsa_circ_0000437 upregulates and promotes disease progression in rheumatic valvular heart disease

**DOI:** 10.1002/jcla.24197

**Published:** 2021-12-24

**Authors:** Linwen Zhu, Zhifang Wang, Lebo Sun, Dawei Zheng, Bingchuan Hu, Ni Li, Guofeng Shao

**Affiliations:** ^1^ Department of Cardiothoracic Surgery Ningbo Medical Centre Lihuili Hospital Ningbo University Ningbo China; ^2^ Medical School of Ningbo University Ningbo China; ^3^ Institute of Pharmaceutics College of Pharmaceutical Sciences Zhejiang University Hangzhou China

**Keywords:** biological function, biomarker, has_circ_0000437, rheumatic valvular heart disease (RVHD)

## Abstract

**Background:**

Currently, the diagnosis and outcome of rheumatic valvular heart disease (RVHD) are less than ideal, and there are no accurate biomarkers. Circular RNA (circRNA) might participate in the occurrence and development of RVHD.

**Materials and methods:**

We use circRNA microarray to filter out the target has_circ_0000437. qRT‐PCR was used to measure the expression levels of hsa_circ_0000437 in RVHD plasma samples. We assessed the diagnostic value of hsa_circ_0000437 in RVHD. Cell function in *vitro* experiment was to explore the effect of has_circ_0000437 on RVHD.

**Results:**

Has_circ_0000437 is highly expressed in RVHD (*p* < 0.001). has_circ_0000437 has the diagnostic value in RVHD. In RVHD, hsa_circ_0000437 can promote cell proliferation and migration but inhibits its apoptosis. This may be due to the combination of has_circ_0000437 and target miRNA in the cytoplasm that affects the progress of RVHD.

**Conclusions:**

Has_circ_0000437 can promote the process of RVHD and may be a potential for the diagnosis and treatment of RVHD.

## Graphical abstract

Apoptotic cell rate of **hVICs** cells following downregulation of hsa_circ_0000437. (B) Apoptotic cell rate of HVICS cells following upregulation of hsa_circ_0000437. (C) Cell‐cycle distribution of **hVICs** cells following downregulation of hsa_circ_0000437. (D) Cell‐cycle distribution of **hVICs** cells following upregulation of hsa_circ_0000437. NC, negative control; KO, knockout; *n* = 3, * *p* < 0.05, *** *p* < 0.001.

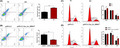

## 1 INTRODUCTION

Rheumatic heart disease (RHD) is a type of acute rheumatic fever (ARF), an autoimmune disorder triggered by group A β‐hemolytic streptococcus infection; recurrent ARF causes permanent valve disease.^1^, ^2^ Although the incidence of RHD is declining globally, it maintains a high incidence in low‐ and middle‐income countries such as in Africa.^3^ At present, rheumatic valvular heart disease (RVHD) is a significant cause of the need for cardiac surgery in China, accounting for 40% of heart diseases. RVHD usually affects the left side of the heart, primarily the mitral valve (MV), and some cases also involve the aortic valve (AV); isolated AV lesions are rare.^4^ During the progression of RVHD, ARF may recur and cause progressive damage to these valves. Combined with hemodynamic changes, the disease process aggravates fibrosis and proliferation of the valve tissue. Heart failure (HF) and atrial fibrillation (AF) appear in the later stages of the disease.^5^ Because the progression of RVHD is insidious, and the typical clinical symptoms of first ARF are rare, there is often apparent valve disease upon diagnosis of RVHD.^6^ More than 50% of RVHD cases require heart valve surgery. Patients with mechanical valve replacement require life‐long anticoagulation therapy after surgery, all of which severely affect the quality of life.^1^ At present, the essential examination method for RVHD is transthoracic echocardiography (TTE); however, the detection accuracy of TTE is affected by the operator, and the technique is prone to errors. The test indicators that assist in diagnosing RVHD include erythrocyte sedimentation rate, C‐reactive protein, mucin, and protein electrophoresis. There are also immune indicators such as circulating immune complexes, total serum complement and complement C3, immunoglobulins IgG, IgM, IgA, B lymphocyte detection, and anti‐myocardial antibodies.^7^, ^8^ The sensitivity and specificity of these tests are not high, and they may not remain elevated during the chronic phase of the disease. Therefore, it is critical to identify biomarkers of RVHD, clarify the biological function and mechanism of RVHD valve disease, and provide adequate tools for the diagnosis and treatment of RVHD.

Circular RNA (circRNA) is a circular molecule formed by lasso‐driven circularization or circularization driven by intron pairing.^9^ On the other hand, circRNAs can be divided into noncoding circRNAs and coding circRNAs.^10^, ^11^ Unlike linear RNA, circRNA has a stable circular structure, so nucleases do not quickly degrade it. Although the earliest circRNAs were found in plant viruses in 1976, they were considered byproducts of transcription and were mistakenly believed to have no critical biological functions.^12^ However, recent studies have shown that circRNAs regulate gene expression in a variety of ways. The biological functions of circRNA include miRNA molecular sponges, regulation of gene transcription, interaction with RNA‐binding protein, and translating proteins.^13^, ^14^, ^15^, ^16^, ^17^ RNA‐seq data shows that more than 1,000 types of circRNA have been detected in adult tissues and blood samples such as the colon, heart, kidney, liver, lung, stomach, and brain. Most circRNA has tissue and blood specimen specificity, and circRNA is involved in regulating these diseases.^18^, ^19^, ^20^, ^21^, ^22^ Therefore, in combination with these characteristics, circRNA might help detect disease occurrence, development, diagnosis, and treatment.

There are many studies on circRNA in cardiovascular diseases (CVD) such as coronary heart disease, hypertension, atherosclerosis, and cardiomyopathy.^23^, ^24^, ^25^, ^26^ However, there are few studies of circRNA in RVHD, and it is urgent to explore its role in RVHD. For this reason, it is critical to identify effective test indicators for early disease prediction, outcome prediction, diagnosis, and treatment. In the present study, the expression profile of circRNA in the plasma of three RVHD patients and three control patients was analyzed using circRNA microarrays. Differentially expressed circRNA was identified (GSE168932, https://www.ncbi.nlm.nih.gov/geo/query/acc.cgi?acc=GSE168932)when fold change >2 and *p* < 0.05. We combined circBase and GEO database to identify the target circRNA has_circ_0000437 that was not previously studied in the literature and then expand the sample size to verify the expression of the target has_circ_0000437 and explore its possibility as a biomarker for RVHD. The influence of has_circ_0000437 on the progression of RVHD was also explored using in vitro experiments.

## 2 MATERIALS AND METHODS

### 2.1 Clinical specimens

From January 2019 to December 2019, 42 RVHD patients and 42 NRVD patients were hospitalized in the Department of Cardiothoracic Surgery of Ningbo Medical Treatment Center, Li Huili Hospital. Pulmonary artery systolic pressure (PASP) was graded according to the TTE tricuspid regurgitation method. The grading standard was as follows: normal <30 mmHg, mild 30–50 mmHg, moderate 51–70 mmHg, and severe ≥71 mmHg.^27^ Multivalvular disease refers to more than two valves’ combined disease, including MV, AV, and tricuspid valve. Coronary angiography was performed for patients over 50 years of age with suspected coronary heart disease.

Inclusion criteria were as follows: RVHD and NRVD diagnosed with clinical symptoms, physical examination, electrocardiograph (ECG), chest X‐ray, and TTE; erythrocyte sedimentation rate and anti‐streptolysin O (ASO) normal, and rheumatic activity period excluded; recent acute HF attacks; and other complications. We selected 42 healthy people with normal physical examinations in our hospital as the control group, and TTE was included in the screening project. The clinicopathological data of all subjects are shown in Table S1. The ethics committee of Ningbo Medical Treatment Center, Li Huili Hospital, approved all research protocols (IRB No. KY2020PJ141).

### 2.2 Collection and storage of plasma samples

We drew 3 ml of peripheral venous blood into EDTA‐containing anticoagulation tubes, centrifuged at 3000 rpm for 15 min, removed the upper light yellow plasma layer in 2 ml RNase‐free eppendorf tubes, and stored the samples at −80°C until use.

### 2.3 CircRNA microarray assays

We used Arraystar Human circRNA microarray (Arraystar) to test 3 pairs of RVHD and normal control samples. We analyzed and processed the detected data with R software package and uploaded the original data to the GEO database. Finally, circRNAs with a fold change of >2.0 and *p*‐value <0.05 were selected as candidates for subsequent research.

### 2.4 Total RNA extraction and reverse transcription

According to the manufacturer's instructions, the total RNA in plasma was extracted with TRIzol LS reagent (Invitrogen). We then used a DS‐11 spectrophotometer machine (DeNovix, Germany, USA) to measure the quality and concentration of total RNA. The ratio of A260/A280 absorbance was used to evaluate the RNA quality. For qualified samples, the absorbance range was between 1.8 and 2.1. We used the reverse transcription GoScript RT system (Promega) to synthesize cDNA with total RNA and random primers according to the manufacturer's instructions.

### 2.5 qRT‐PCR

We used the Mx3005P qRT‐PCR PCR system (Stratagene) to amplify hsa_circ_0000437 GoTaq using qPCR Master Mix (Promega) according to the kit instructions. The specific convergent primer sequences of hsa_circ_0000437 are shown in Table S2. We use glyceraldehyde 3‐phosphate dehydrogenase (GAPDH) as an external reference, and its primer sequence is shown in Table S2. The primers were synthesized by BGI tech. We expressed data using 2^−ΔCq^, which is directly proportional to expression data.

### 2.6 Cell culture and transfection

The immortalized primary cell hVICs cell line constructed from the heart valve interstitium of patients with RVHD was purchased from iCell (iCell,). hVICs cell line was cultured in basic medium for primary mesenchymal cells (iCell,), containing 1% primary mesenchymal cell culture additive (iCell,) and 10% fetal bovine serum (Gibco) and 1% penicillin/streptomycin (Life Technologies). The cells were cultured in an incubator (Thermo Fisher) containing 5% CO_2_ at 37°C. Sequences of has_circ_0000437 small interfering RNA (siRNA) (GenePharma,) and siRNA control RNA (siRNA negative control (NC)) are shown in Table S3. Empty pGL3 vector and empty pcDNA3.1 vector overexpression has_circ_0000437 recombinant plasmid (Geneseed Biotech Co., Ltd.,) were transferred into hVICs in the logarithmic growth phase. The cells were transfected with Lipofectamine 2000 (Invitrogen,) according to the manufacturer's protocol.

### 2.7 Cell counting kit 8 (CCK‐8) assay

The transfected cells were seeded into 96‐well plates at various times (1, 2, 3, 4, and 5 days) at 5 × 10^3^ per well. Then, 10 μl of CCK‐8 reagent (Dojindo,) was added to each well, and cells were incubated at 37°C for 3 h. Finally, a SpectraMax M5 Microplate Reader (Molecular Devices) was used to measure the absorbance (OD) value at 450 nm to determine the degree of cell growth.

### 2.8 Wound healing assay

After the transfected cells are cultured to confluence, we scraped the cells with a 200 µl pipette tip to create an artificial wound. We washed the cells with phosphate‐buffered saline (PBS) to remove floating cells, obtained photographs at 0 h, and then again at 48 h. Image Pro Plus v6.0 software package (Media Cybernetics Inc.,) was used to measure the distance of cell migration.

### 2.9 Transwell migration assay

We used 24‐well Transwell chambers (Costar,) to estimate cell migration. We collected transfected cells and resuspended them in a serum‐free medium. We then seeded 200 µl of 8 × 10^4^ cells into the upper chamber. We added 500 µl of medium containing 20% FBS to the lower chamber. After incubating for 24 h, cells were fixed with 4% paraformaldehyde, then stained with 0.1% crystal violet, and finally counted at 40× magnification with an optical microscope.

### 2.10 Cell apoptosis assay

The transfected cells were removed using EDTA‐free trypsin, and the cells were resuspended in binding buffer. According to the instruction manual, cells were stored at room temperature, stained with Annexin V‐FITC/PI Apoptosis Kit (Multi Sciences, China) according to the instruction manual, and stored in the dark for 15 min. A FACSCalibur flow cytometer (Becton Dickinson Co.,) was used to identify apoptotic cells.

### 2.11 Cell‐cycle assay

First, we starved cells with a serum‐free medium to synchronize the cell cycle. We collected the transfected cells, washed them in PBS, and fixed them in 70% ethanol at –20°C overnight. We washed the cells with precooled PBS, added 1 ml PI /RNase staining buffer (Multi Sciences,) for staining, incubated for 30 min in the dark, and measured the cell cycle fraction using the FACSCalibur flow cytometer (Becton Dickinson Co.,).

### 2.12 FISH assay

The FISH probe used a specific probe targeting the has_circ_0000437 sequence (RiboBio,), namely the cy5 labeled has_circ_0000437 probe. We stained cell nuclei using 4’,6‐diamidino‐2‐phenylindole (DAPI). All steps were performed according to the manufacturer's instructions (RiboBio,), and the fluorescence images were observed through a microscope (Leica Microsystems,).

### 2.13 Statistical analysis

The data were analyzed using Statistical Program for Social Sciences 20.0 software (SPSS, IBM, USA), and the data were expressed as the mean ± standard deviation. We used the two‐sided Student's *t* test to calculate the differences between groups. *p* < 0.05 was considered statistically different.

## 3 RESULTS

### 3.1 Characterization and specific primers of hsa_circ_0000437

**FIGURE 1:**
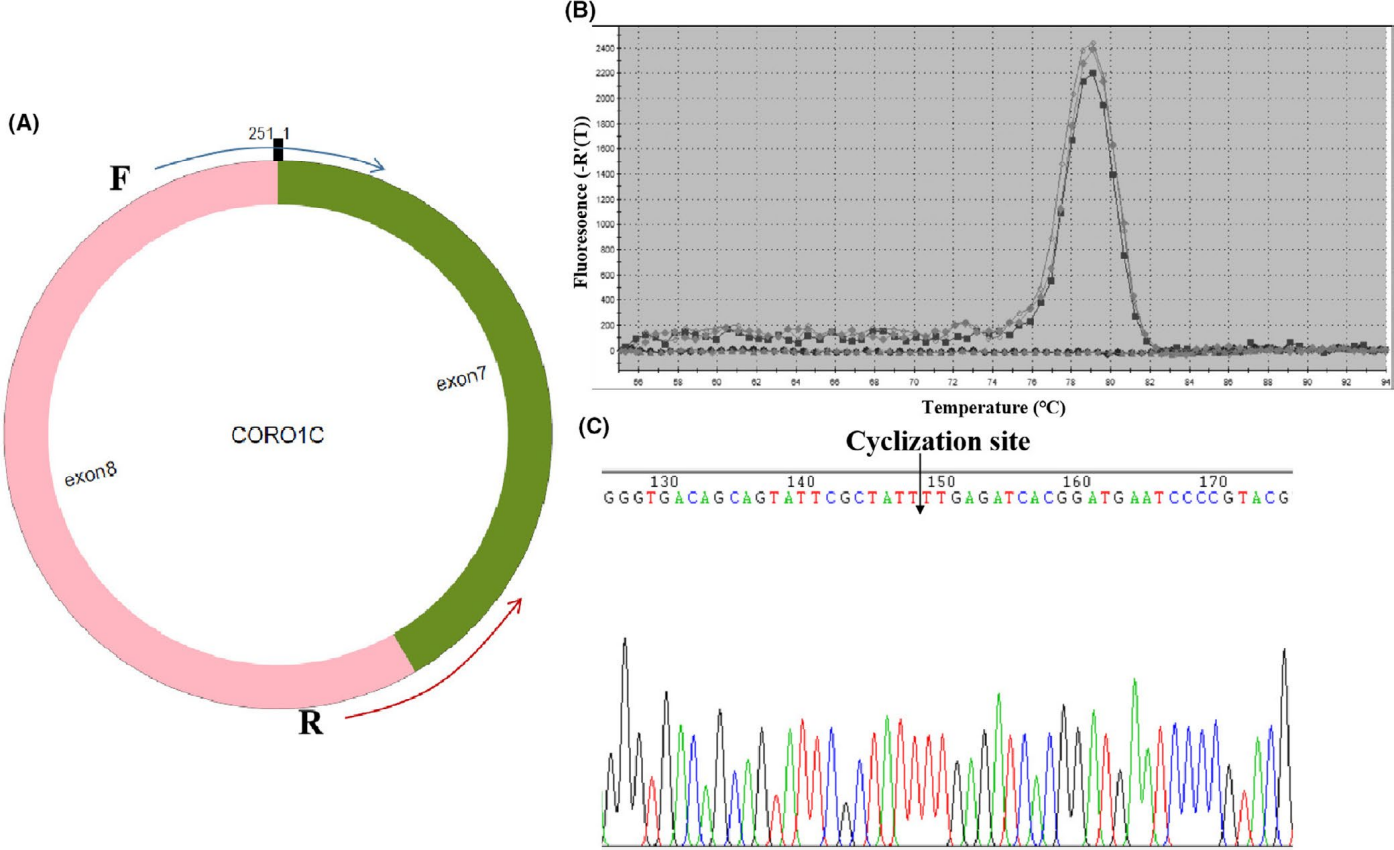
Amplification of hsa_circ_0000437. (A) hsa_circ_0000437 and divergent primers. hsa_circ_0000437 comes from exon 7 and exon 8 of CORO1C, 251 nucleotides in length. The forward primer contains the splice site sequence. (B) Melting curve analysis of the qRT‐PCR products of hsa_circ_0000437. Three representative samples are shown. (C) Sanger sequencing of hsa_circ_0000437. The arrow shows the site of cyclization

Hsa_circ_0000437 was encoded from the 12q24.11 region. In this chromosomal region, the typical transcript is coronin 1C (CORO1C) mRNA, consisting of 19 exons. hsa_circ_0000437 is composed of exon 7 to exon 8 (Figure 1A). We amplified hsa_circ_0000437 by designing specific convergent hsa_circ_0000437 primers. To verify the correctness of the qRT‐PCR primers, we first obtained melting curves. We found that the amplified product produced only one peak (Figure 1B), suggesting no nonspecific amplification and no primer dimers. The qRT‐PCR product was also analyzed using the Sanger sequencing method. We found that the sequence of the product after hsa_circ_0000437 amplified by qRT‐PCR contained a circularization site (Figure 1C). This sequence was precisely the same as listed in hsa_circ_0000437 on the circBase website (http://circrna.org/).

### 3.2 Hsa_circ_0000437 is increased in the plasma of RVHD patients relative to NRVD and normal controls

**FIGURE 2:**
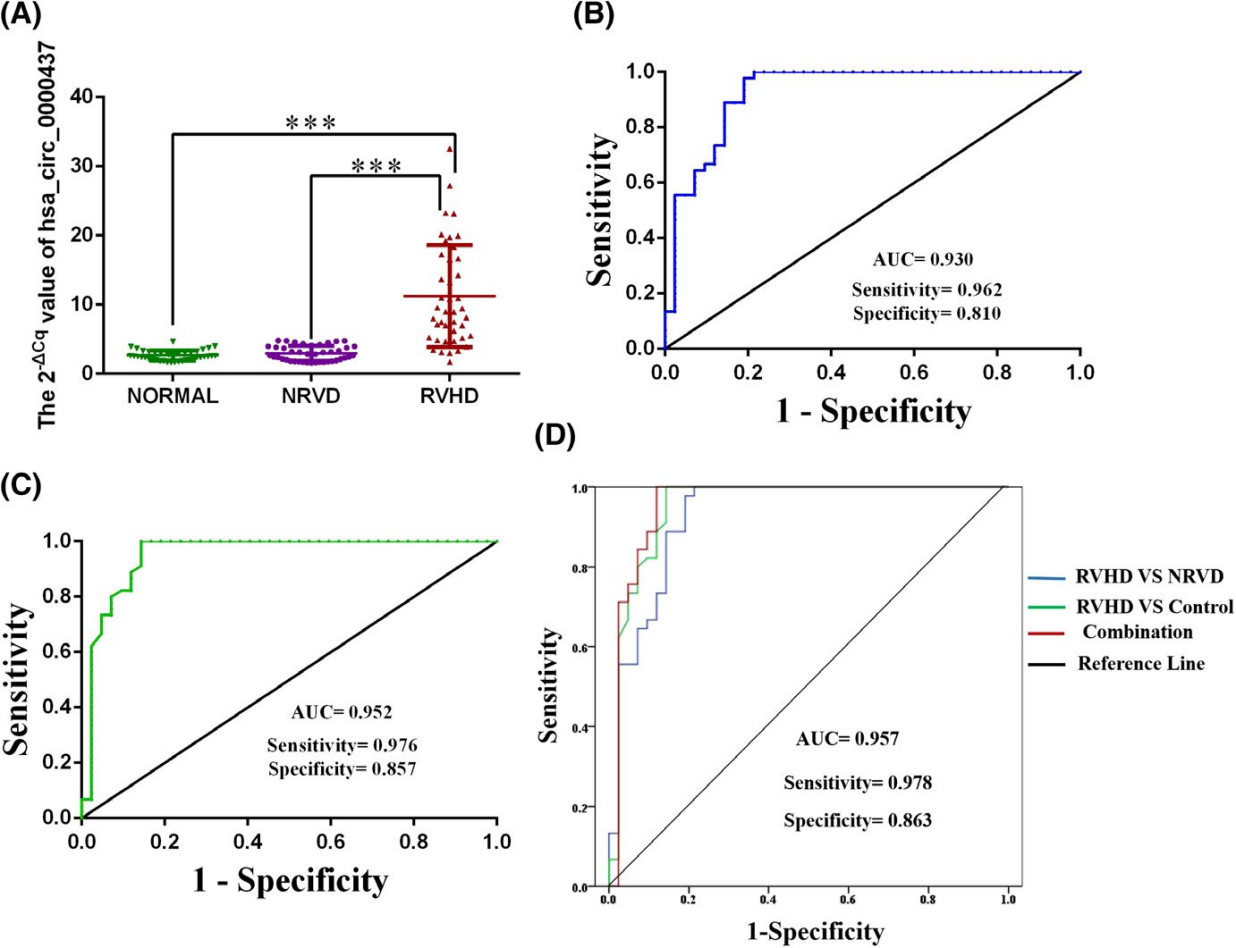
Expression levels of hsa_circ_0000437 in RVHD plasma samples and their diagnostic value. (A) Expression levels of hsa_circ_0000437 in the RVHD group were significantly higher than those in the NRVD and the normal control groups (*n* = 42). (B) AUC of the RVHD group compared with the NRVD group. (C) AUC of the RVHD group compared with the normal control group. (D) AUC after the combination of the RVHD, NRVD, and normal control groups. AUC: the area under the curve, ***p < 0.001

We measured the expression levels of hsa_circ_0000437 in plasma samples of 42 RVHD patients and 42 NRVD patients and normal controls. We found that the levels of hsa_circ_0000437 were significantly higher in RVHD patients than in normal controls and NRVD patients (Figure 2A), consistent with the results of the microarray analysis.

We also used multiclass logistic regression analysis to compare the expression levels of has_circ_0000437 in the RVHD, NRVD, and control groups. There was no statistically significant difference in the expression levels of has_circ_0000437 between the NRVD and control groups (odds ratio [OR] = 0.771, 95% CI: 0.335–1.774, *p* = 0.54) (Table S4). The expression levels of has_circ_0000437 between the RVHD and the control groups were significantly different (OR = 0.024, 95% CI: 0.005–0.106, *p* < 0.001) (Table S4). The expression levels of has_circ_0000437 were significantly different between the RVHD and NRVD groups (OR = 0.031, 95% CI: 0.007–0.132, *p* < 0.001) (Table S5).

### 3.3 Relationship between the expression level of has_circ_0000437 and clinicopathological factors

The Spearman and Pearson correlation analysis methods were used to analyze the correlation between the expression levels of has_circ_0000437 and clinical parameters. Expression of has_circ_0000437 correlated with multivalvular disease (*p* = 0.013). Because the MV is diseased in virtually all patients with RVHD, it was impossible to simply analyze its correlation with circRNA expression levels (Table 1).

**TABLE 1:** Correlation between the hsa_circ_0000437 expression level and clinical parameters

Clinical parameters	Correlation coefficient	*p*
Gender (femal/male)	0.041	0.798
Age (year)	0.160	0.313
Smoking (yes/no)	−0.040	0.804
Drinking (yes/no)	0.152	0.338
Type 2 diabetes (yes/no)	0.148	0.349
Hypertension (yes/no)	0.129	0.415
Height (cm)	0.137	0.386
Weight (kg)	0.029	0.854
BMI (kg/m2)	0.023	0.885
LVEDD (mm)	−0.096	0.546
LAD (mm)	0.116	0.466
IVS (mm)	0.013	0.937
LVEF (%)	0.055	0.730
PASP (mmHg)	0.179	0.256
Pulmonary artery pressure rating (normal/mild/moderate/severe)	0.045	0.775
Heart function classification (Ⅰ/Ⅱ/Ⅲ/Ⅳ)	−0.192	0.224
Atrial fibrillation (yes/no)	0.096	0.544
Mitral valve disease (yes/no)	–	–
Aortic valve disease (yes/no)	−0.172	0.276
Tricuspid valve disease (yes/no)	0.142	0.371
Whether multivalvular disease (yes/no)	0.380	0.013

Abbreviations: BMI, Body mass index; LVEDD, Left ventricular end‐diastolic diameter; LAD, Left atrial diameter; IVS, Interventricular septum; LVEF, Left ventricular ejection fraction; PASP, Pulmonary artery systolic pressure.

### 3.4 Relationship between has_circ_0000437 and RVHD

We analyzed the relationship between has_circ_0000437 and RVHD by combining the RVHD and NRVD groups’ clinical parameters. Single‐factor logistic regression analysis showed that has_circ_0000437 correlated with RVHD (OR = 0.053, 95% CI: 0.014–0.195, *p* < 0.001). Multivariate logistic regression analysis showed that, after adjusting for all confounding factors, has_circ_0000437 remained associated with RVHD (OR = 0.018, 95% CI: 0.001–0.283, *p* < 0.001) (Tables 2,3).

**TABLE 2:** Model definition of the RVHD group compared to the NRVD group

Model	Correction variable
Model 1	No correction
Model 2	Age, gender, and BMI
Model 3	Age, gender, BMI, smoking, and drinking
Model 4	Age, gender, BMI, smoking, drinking, high blood pressure, and diabetes
Model 5	Age, gender, BMI, smoking, drinking, hypertension, diabetes, left ventricular end‐diastolic diameter, left atrial inner diameter, ventricular septal thickness, and EF
Model 6	Age, gender, BMI, smoking, drinking, high blood pressure, diabetes, left ventricular end‐diastolic diameter, left atrial diameter, ventricular septal thickness, EF, pulmonary artery systolic pressure, pulmonary artery pressure grade, cardiac function classification, and atrial fibrillation, whether it is polymembranous disease

**TABLE 3:** Single‐factor and multivariate logistic regression analysis results of the RVHD group compared to the NRVD group

Model	OR	95% CI		*p*
		Lower limit	Upper limit	
Model 1	0.053	0.014	0.195	<0.001
Model 2	0.051	0.012	0.211	<0.001
Model 3	0.037	0.007	0.188	<0.001
Model 4	0.036	0.007	0.187	<0.001
Model 5	0.037	0.006	0.237	<0.001
Model 6	0.018	0.001	0.283	<0.001

We analyzed the relationship between has_circ_0000437 and RVHD by combining age and gender of the RVHD and the control groups. Univariate logistic regression analysis showed that has_circ_0000437 correlated with RVHD (OR = 0.021, 95% CI: 0.003–0.128, *p* < 0.001). After adjusting for age and gender factors, has_circ_0000437 remained associated with RVHD (OR = 0.016, 95% CI: 0.002–0.124, *p* < 0.001) (Tables 4,5). These results suggest that has_circ_0000437 is an independent risk factor for RVHD.

**TABLE 4:** Model definition of the RVHD group compared to the control group

Model	Correction variable
Model 1	No correction
Model 2	Age, gender

**TABLE 5:** Single‐factor and multivariate logistic regression analysis results of the RVHD group compared to the control group

Model	OR	95% CI		*p*
		Lower limit	Upper limit	
Model 1	0.021	0.003	0.128	<0.001
Model 2	0.016	0.002	0.124	<0.001

### 3.5 Diagnostic efficacy of has_circ_0000437 for RVHD

ROC curve analysis was used to determine the diagnostic efficacy of has_circ_0000437 for RVHD. The area under the ROC curve of the RVHD group compared with the NRVD group was 0.930 (*p* < 0.001). The sensitivity was 0.962, and the specificity was 0.810 (Figure 2B). The area under the curve (AUC) of the RVHD group compared with the normal control group was 0.952 (*p* < 0.001). The sensitivity was 0.976, and the specificity was 0.857 (Figure 2C). The combined AUC was 0.957 (*p* < 0.001). The sensitivity was 0.978, and the specificity was 0.863 (Figure 2D). We also compared the diagnostic efficacy of electrocardiogram (ECG) and TTE with has_circ_0000437 (Table 6). The sensitivities of the two were 0.70 and 0.62, respectively, and the specificities were 0.62 and 0.74, respectively.^28^ The diagnostic efficiency of has_circ_0000437 in the RVHD group compared with the NRVD and the normal control groups were higher than that of ECG and TTE.

**TABLE 6:** Diagnostic values of has_circ_0000437 and two traditional RHD markers

RVHD group VS. control group	Sensitivity	Specificity
Has_circ_0000437	0.976	0.857
ECG	0.70	0.62
TTE	0.62	0.74

### 3.6 Hsa_circ_0000437 promotes proliferation of hVICs cells

**FIGURE 3:**
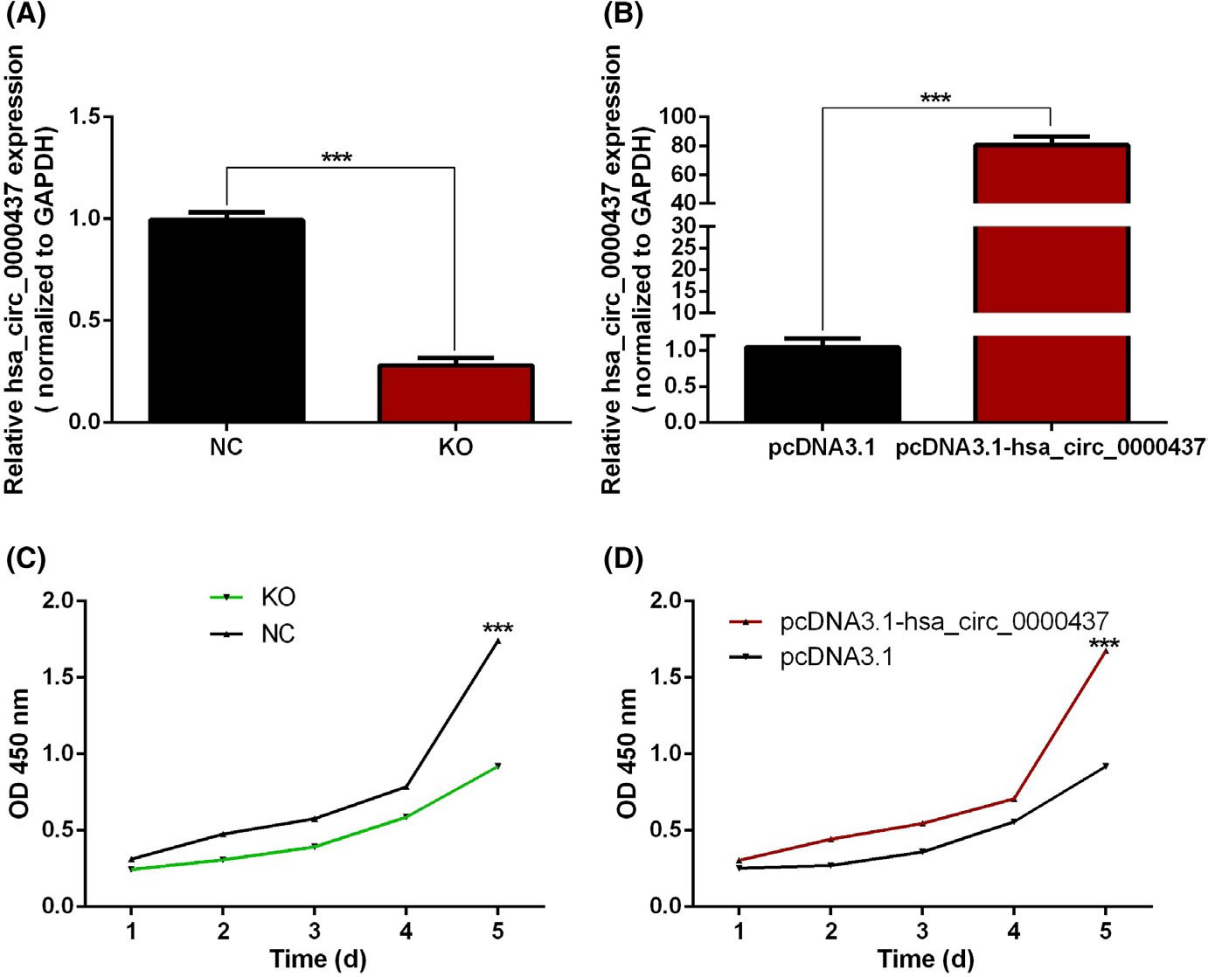
Expression levels of hsa_circ_0000437 after upregulation and downregulation, and effects of hsa_circ_0000437 on hVICs proliferation. (A) siRNA downregulation effect of hsa_circ_0000437 in hVICs cells. (B) Upregulation effect of hsa_circ_0000437 overexpression plasmid in hVICs. (C) Grow curve of hVICs cells following downregulation of hsa_circ_0000437. (D) Grow curve of hVICs cells following upregulation of hsa_circ_0000437. NC, negative control; KO, knockout; *n* = 3, ***p < 0.001

Hsa_circ_0000437 overexpression plasmid and blank negative control (NC) vector pcDNA3.1 were transfected into hVICs cells. siRNA and NC of hsa_circ_0000437 were also transfected into hVICs cells and were found to upregulate and downregulate the expression levels of hsa_circ_0000437 in hVICs cells (Figure 3A,B). Using the CCK‐8 assay, we found that hsa_circ_0000437 siRNA significantly inhibited the proliferation of hVICs cells (Figure 3C). When hsa_circ_0000437 was overexpressed, it promoted the proliferation of hVICs (Figure 3D).

### 3.7 Hsa_circ_0000437 promotes migration of hVICs cells

**FIGURE 4:**
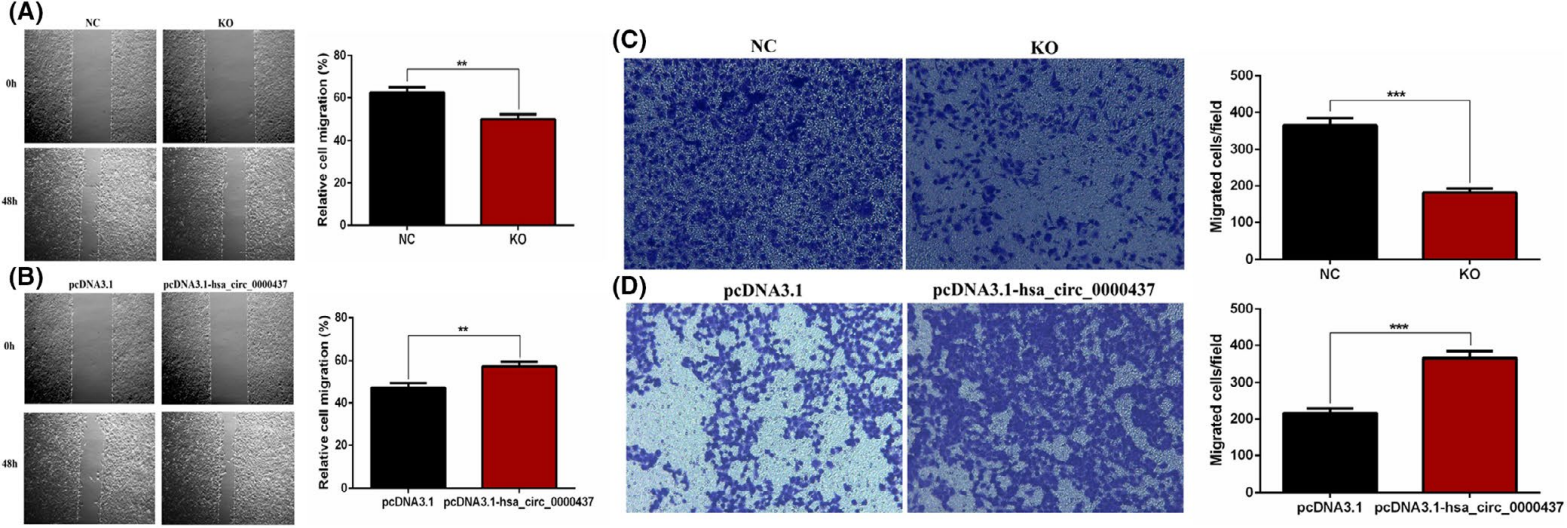
Effects of hsa_circ_0000437 on hVICs migration. (A) Relative cell migration of hVICs following downregulation of hsa_circ_0000437. (B) Relative cell migration of hVICs following upregulation of hsa_circ_0000437. (C) Cell migration numbers of hVICs following downregulation of hsa_circ_0000437. (D) Cell migration numbers of hVICs following upregulation of hsa_circ_0000437. NC, negative control; KO, knockout; *n* = 3, **p < 0.01, ***p < 0.001

Wound healing assay revealed that hsa_circ_0000437 siRNA significantly reduced cell migration (Figure 4A). When hsa_circ_0000437 was overexpressed, cell migration was increased (Figure 4B). The Transwell assay revealed that hsa_circ_0000437 siRNA significantly reduced the number of cells migrating to the lower chamber (Figure 4C). When hsa_circ_0000437 was overexpressed, the number of cells migrating to the lower chamber increased (Figure 4D). These findings suggest that hsa_circ_0000437 promotes the migration ability of hVICs.

### 3.8 Hsa_circ_0000437 inhibits apoptosis and affects cell cycle in hVICs cells

**FIGURE 5:**
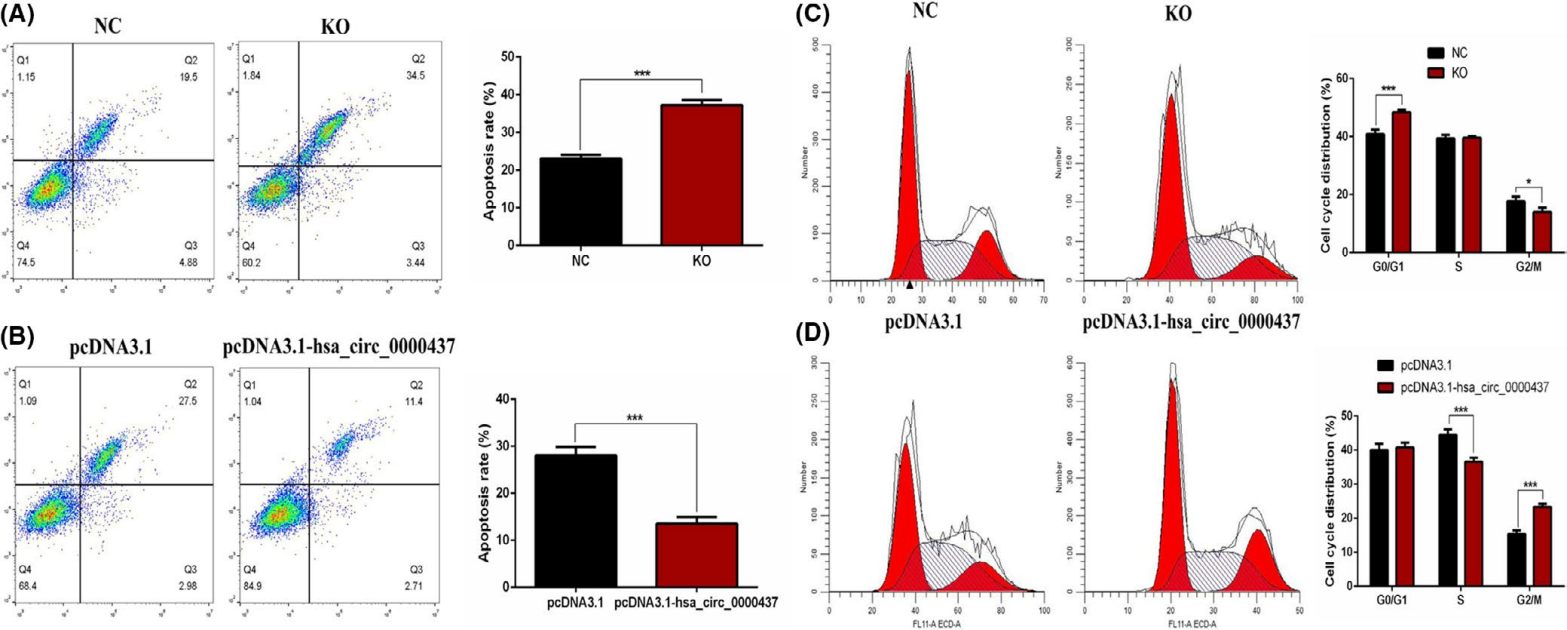
Effects of hsa_circ_0000437 on hVICs apoptosis and cell‐cycle distribution. (A) The apoptosis rate of hVICs following downregulation of hsa_circ_0000437. (B) The apoptosis rate of hVICs following upregulation of hsa_circ_0000437. NC, negative control; KO, knockout; *n* = 3, ***p < 0.001. (C) Cell‐cycle distribution of hVICs following downregulation of hsa_circ_0000437. (D) Cell‐cycle distribution of hVICs following upregulation of hsa_circ_0000437. NC, negative control; KO, knockout; *n* = 3, *p < 0.05, ***p < 0.001

Using flow cytometry analysis, we found that hsa_circ_0000437 siRNA significantly increased the number of apoptotic cells (Figure 5A). When hsa_circ_0000437 was overexpressed, the number of apoptotic cells was reduced (Figure 5B). These findings suggest hsa_circ_0000437 inhibits apoptosis in hVICs.

Also using flow cytometry analysis, we found that hsa_circ_0000437 siRNA blocked the cell cycle in the G0/G1 phase (Figure 5C). When hsa_circ_0000437 was overexpressed, the cell cycle was blocked in G2/M phases (Figure 5D). These findings suggest that hsa_circ_0000437 promoted cell cycle progression.

### 3.9 Positioning and sponge function of hsa_circ_0000437

**FIGURE 6:**
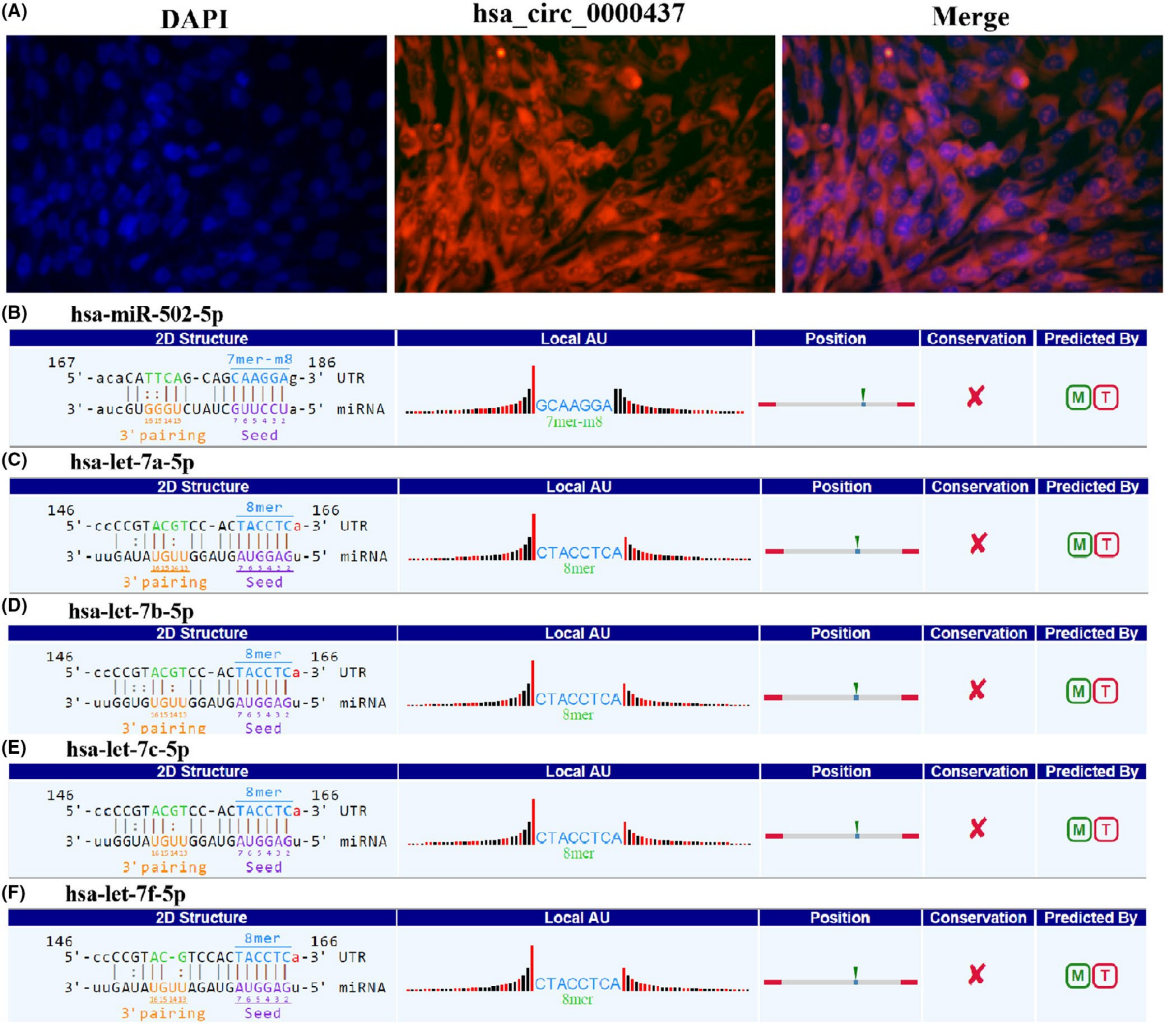
miRNA sponge effect of hsa_circ_0000437. (A) FISH experiments confirming that hsa_circ_0000437 is located in the cytoplasm. The nucleus is labeled with DAPI, and hsa_circ_0000437 is labeled with Cy3. Three sets of experiments were performed. (B) Bioinformatics prediction of the combination of hsa_circ_0000437 and hsa‐miR‐502‐5p. (C) Bioinformatics prediction of the combination of hsa_circ_0000437 and hsa‐let‐7a‐5p. (D) Bioinformatics prediction of the combination of hsa_circ_0000437 and hsa‐let‐7b‐5p. (E) Bioinformatics prediction of the combination of hsa_circ_0000437 and hsa‐let‐7c‐5p. (F) Bioinformatics prediction of the combination of hsa_circ_0000437 and hsa‐let‐7f‐5p

The location of hsa_circ_0000437 in hVICs cells was confirmed using FISH. We found that hsa_circ_0000437 (red fluorescence) was located in the cytoplasm (Figure 6A). Based on these results, we conclude that hsa_circ_0000437 is a circRNA from the CORO1C locus, located in the cytoplasm. We used circbank and circular RNA interaction software to predict the miRNAs bound to hsa_circ_0000437. After taking the intersection, we found that they were hsa‐miR‐502‐5p, hsa‐let‐7a‐5p, hsa‐let‐7b‐5p, hsa‐let‐7c‐5p, and hsa‐let‐7f‐5p (Figure 6B–F). These miRNAs may co‐localize with hsa_circ_0000437 and act as sponges in the cytoplasm.

## 4 DISCUSSION

Many studies on circRNA have shown that these molecules participate in the occurrence and development of various diseases. Using circRNA microarray screening, Zuo et al. found that circPDS5B and circCDC14A were differentially expressed and upregulated in patients with acute ischemic stroke.^29^ Han et al. found that silencing hsa_circ_0071036 inhibited tumor growth in patients with pancreatic cancer. The mechanistic analysis showed that hsa_circ_0071036 acted as an miR‐489 sponge; the authors found that the abnormal expression of hsa_circ_0071036 was related to poor pathological features and outcome.^30^ Pan et al. used a mouse lung cancer model to find that circFLNA promoted the proliferation, migration, and invasion of lung cancer cells through the sponge effect of miR‐486‐3p; the circFLNA‐miR‐486‐3p‐XRCC1/CYP1A1 regulatory axis was established through subsequent mechanistic experiments.^31^ Gao et al. screened the plasma of patients with persistent atrial fibrillation by circRNA microarray and found that hsa_circ_0004104 was significantly downregulated; hsa_circ_0004104 negatively correlated with TGF‐β1, suggesting the potential of hsa_circ_0004104 as a biomarker for persistent AF.^32^

These studies on circRNA focused on tumors and cardiovascular and cerebrovascular aspects; few studies of valvular heart disease exist. Yu et al. found that circTGFBR2 acted as a sponge for miR‐25‐3p in calcified aortic valves, inhibiting the expression of miR‐25‐3p and establishing the circTGFBR2‐miR‐25‐3p‐TWIST1 regulatory axis; these findings suggested that circTGFBR2 participates in AV calcification.^33^ However, we could not identify studies of the expression of circRNA in RVHD. Therefore, we performed the present study to determine the expression profile of circRNA in RVHD, explore the potential pathogenic function and mechanism of target circRNA in diseases, and identify diagnostic markers and treatment targets.

We used circRNA microarray to screen the differentially expressed circRNA in the plasma of RVHD and control and combined circBase and GEO databases to identify the target hsa_circ_0000437. The expression levels of has_circ_0000437 in a large sample were higher in RVHD patients, consistent with the results of the circRNA microarray. Expression of has_circ_0000437 of the RVHD group was significantly different from that of the NRVD group. Multivariate logistic regression analysis was used to compare the expression levels of has_circ_0000437 between RVHD, NRVD, and the control group; we found that has_circ_0000437 was a significantly independent influencing factor of RVHD. We also found no significant difference in the expression of has_circ_0000437 between the NRVD and control groups, suggesting that has_circ_0000437 is independent of RVHD and its underlying pathogenesis is different from NRVD. We found that the expression of has_circ_0000437 was negatively correlated with multivalvular disease. A search of the relevant literature revealed no difference between the pathogenesis of single valve and multivalvular disease in RVHD.

Combining single‐factor and multivariate logistic analysis of clinical parameters, we found that has_circ_0000437 was an independent risk factor for RVHD. Currently, examination methods for diagnosing RVHD include ECG, chest X‐ray, TTE, TEE, and cardiac catheterization tests (guidelines). TEE and cardiac catheterization tests are associated with complications and cost issues related to inspections. We found that the high sensitivity and specificity of has_circ_0000437 in plasma and AUC suggest that has_circ_0000437 may be a biomarker for the diagnosis of RVHD, characterized by convenience and relatively lower cost.

The role of miRNA sponges in the biological function of circRNA is an intense area of research. MiRNA is an essential regulator of gene expression that targets mRNA to prevent its translation or promote its degradation. There are several miRNA‐binding sites on circRNA. In the cytoplasm, they bind miRNA to inhibit miRNA activity and enhance the expression of target genes.^34^ In the present study, we found that has_circ_0000437 affected the proliferation, migration, cell cycle, and apoptosis of hVICs. The biological function of hVICs was likely affected by the joint action of sponge miRNA. Hui et al. conducted an miRNA microarray analysis on calcified aortic valve tissue. They found that the Let7 family (let‐7a, let‐7b, let‐7c, let‐7d, let‐7e, let‐7f, and let‐7g) was downregulated in valve tissue, suggesting that this family may be involved in the calcification of the valve.^35^ Shu et al. reported that IGF‐1–IGF1R–hsa‐let‐7c‐5p is involved in osteogenic differentiation in mesenchymal cells. IGF‐1 promotes the proliferation of fibroblasts and then causes the deposition of collagen and collagen fibers. It also induces the transformation of endothelial cells into osteoblasts, promotes calcium salt deposition, and ultimately leads to valve hardening and calcification.^36^ Geng et al. found that the expression of hsa‐let‐7f‐5p was downregulated in mesenchymal cells, and it negatively regulated the target gene to promote increased levels of interleukin‐6 (IL‐6).^37^ Studies showed that chronic RVHD might involve a continuous inflammatory response that leads to continuous damage to the valve and aggravates the development of the disease. In chronic RVHD patients, there is an increase in some inflammatory indicators in the serum, especially IL‐6, suggesting that it plays a role in chronic valve changes in RVHD.^38^

Based on our findings, has_circ_0000437 can be used as a biomarker for the diagnosis of RVHD. It can also affect the biological function of immortalized primary cell hVICs constructed from heart valve tissue of patients with RVHD. These findings suggest that has_circ_0000437 promotes the progression of RVHD and may participate in the pathogenesis of RVHD through sponge absorption of hsa‐let‐7c‐5p and hsa‐let‐7f‐5p. These findings provide a basis for the discovery of potential therapeutic targets for RVHD. The specific mechanism of has_circ_0000437 to promote the occurrence and progression of RVHD needs to be further explored. Of course, the mechanism that has_circ_0000437 promotes the progress of RVHD has yet to be studied. Has_circ_0000437 may affect the development of RVHD by regulating or targeting binding proteins or RNA. These mechanisms need to be further studied. In addition, we need to use animal experiments to verify our findings. For the feasibility of clinical application, further clinical trials and ethical approval are needed.

## CONFLICT OF INTEREST

The authors declare that there is no conflict of interest.

## Supporting information

Table S1‐S5

## Data Availability

The data sets analyzed during the current study are available from the corresponding author upon reasonable request.
