# Supplementary material for: Hsa_circ_0000437 upregulates and promotes disease progression in rheumatic valvular heart disease
Source: J Clin Lab Anal. 2021 Dec 24;36(2):e24197. doi: 10.1002/jcla.24197 (PMC8842158; doi:10.1002/jcla.24197)
Supplement: Supplementary file 1 — Table S1‐S5 [file JCLA-36-e24197-s001.doc]

**Hsa_circ_0000437 up-regulates and promotes disease progression in rheumatic valvular heart disease**

**Supplementary Information**

**Supplementary Table 1. Case characteristics of 42 RVHD patients, 42 NRVD patients, and 42 normal controls.**

**Supplementary Table 2. Primer sequences.**

**Supplementary Table 3. RNA oligonucleotide sequences.**

**Supplementary Table 4.** **Multiple logistic regression with the control group** **as control.**

**Supplementary Table 5. Multivariate logistic regression with the NRVD group as control.**

**Supplementary Table1. Case characteristics of 42 RVHD patients, 42 NRVD patients, and 42 normal controls.**

|  | NORMAL (*n*=42） | RVHD (*n*=42） | NRVD (*n*=42) | *P* |
| --- | --- | --- | --- | --- |
| Age (year) | 59.53±9.98 | 56.38±9.34 | 62.29±9.05 | 0.016 |
| Gender (femal/male) | 28/17 | 33/9 | 20/25 | 0.005 |
| Height (cm) | - | 162.21±7.99 | 164.20±7.90 | 0.157 |
| Weight (kg) | - | 62.69±8.22 | 65.16±11.18 | 0.243 |
| BMI (kg/m^2^) | - | 22.86±2.20 | 24.05±3.15 | 0.043 |
| LVEDD (mm) | - | 45.74±7.13 | 54.38±7.63 | 0.000 |
| LAD (mm) | - | 48.55±8.34 | 41.93±11.15 | 0.000 |
| IVS (mm) | - | 9.82±0.97 | 11.55±1.83 | 0.000 |
| LVEF (%) | - | 64.52±6.16 | 63.62±7.51 | 0.544 |
| PASP (mmHg) | - | 48.55±11.30 | 44.09±11.94 | 0.059 |
| Smoking (yes/no) | - | 7/35 | 12/33 | 0.259 |
| Drinking（yes/no） | - | 6/36 | 7/38 | 0.868 |
| Type 2 diabetes (yes/no) | - | 1/41 | 1/44 | 1.000 |
| Hypertension (yes/no) | - | 6/36 | 24/21 | 0.000 |
| Atrial fibrillation (yes/no) | - | 11/31 | 9/36 | 0.493 |
| Mitral valve disease (yes/no) | - | 42/0 | 25/20 | 0.000 |
| Aortic valve disease (yes/no) | - | 13/29 | 29/16 | 0.002 |
| Tricuspid valve disease (yes/no) | - | 14/28 | 8/37 | 0.095 |
| Whether multivalvular disease (yes/no) | - | 16/26 | 22/23 | 0.310 |
| Heart function classification (Ⅰ/Ⅱ/Ⅲ/Ⅳ） | - | 1/3/34/4 | 0/8/30/7 | 0.264 |
| Pulmonary artery pressure rating  (normal/mild/moderate/severe） | - | 0/33/9/0 | 1/38/5/1 | 0.869 |

Abbreviations: BMI: Body mass index, LVEDD: Left ventricular end diastolic diameter, LAD: Left atrial diameter, IVS: Interventricular septum, LVEF: Left ventricular ejection fraction, PASP: Pulmonary artery systolic pressure.

**Supplementary Table 2. Primer sequences.**

| **Primer** | **Forward primer (5’ to 3’)** | **Reverse primer (5’ to 3’)** |
| --- | --- | --- |
| hsa_circ_0000437(for qPCR) | GGGATGGGTTACATGCCCAA | TCCTGCATATTTTTCTGGCAATCTC |
| GAPDH (for qPCR) | AAGGTGAAGGTCGGAGTCAA | AATGAAGGGGTCATTGATGG |

**Supplementary Table 3. RNA oligonucleotide sequences.**

| **RNA oligos** | **Sequences** |
| --- | --- |
| siRNA-NC  si-hsa_circ_0000437 | Sense: 5’- UUCUCCGAACGUGUCACGUTT -3’  Anti-sense: 5’- ACGUGACACGUUCGGAGAATT -3’  Sense: 5’- GGGACUUGAUGUUAACAAATT -3’  Anti-sense: 5’-UUUGUUAACAUCAAGUCCCTT -3’ |
|  |  |

**Supplementary Table 4. Multiple logistic regression with the control group as control.**

| Group | | Degree of freedom | Significance | OR | 95% CI | |
| --- | --- | --- | --- | --- | --- | --- |
|  |  |  |  |  | Lower limit | Upper limit |
| NRVD | Intercept | 1 | 0.542 | 0.771 | 0.335 | 1.774 |
|  | ΔCq | 1 | 0.540 |  |  |  |
| RVHD | Intercept | 1 | 0.000 | 0.024 | 0.005 | 0.106 |
|  | ΔCq | 1 | 0.000 |  |  |  |

Abbreviations: OR: Odd ratio, 95% CI: 95% confidence interval.

**Supplementary Table 5. Multivariate logistic regression with the NRVD group as control.**

| Group | | Degree of freedom | Significance | OR | 95% CI | |
| --- | --- | --- | --- | --- | --- | --- |
|  |  |  |  |  | Lower limit | Upper limit |
| RVHD | Intercept | 1 | 0.000 | 0.031 | 0.007 | 0.132 |
|  | ΔCq | 1 | 0.000 |  |  |  |
| Control | Intercept | 1 | 0.542 | 1.298 | 0.564 | 2.987 |
|  | ΔCq | 1 | 0.540 |  |  |  |

Abbreviations: OR: Odd ratio, 95% CI: 95% confidence interval.
